# Practical application of a Bayesian network approach to poultry epigenetics and stress

**DOI:** 10.1186/s12859-022-04800-0

**Published:** 2022-07-01

**Authors:** Emiliano A. Videla Rodriguez, Fábio Pértille, Carlos Guerrero-Bosagna, John B. O. Mitchell, Per Jensen, V. Anne Smith

**Affiliations:** 1 School of Biology, University of St Andrews, St Andrews, Fife, KY16 9TH UK; 2 Environmental Toxicology Program, Institute of Organismal Biology, Uppsala University, Uppsala, Sweden; 3 Department of Biomedical & Clinical Sciences (BKV), Linköping University, 58183 Linköping, Sweden; 4 AVIAN Behavioural Genomics and Physiology Group, Department of Physics, Chemistry and Biology, Linköping University, 58183 Linköping, Sweden; 5 EaStCHEM School of Chemistry, University of St Andrews, St Andrews, Fife, KY16 9ST UK

**Keywords:** Bayesian networks, Differential methylation, Epigenetics, Poultry, Stress

## Abstract

**Background:**

Relationships among genetic or epigenetic features can be explored by learning probabilistic networks and unravelling the dependencies among a set of given genetic/epigenetic features. Bayesian networks (BNs) consist of nodes that represent the variables and arcs that represent the probabilistic relationships between the variables. However, practical guidance on how to make choices among the wide array of possibilities in Bayesian network analysis is limited. Our study aimed to apply a BN approach, while clearly laying out our analysis choices as an example for future researchers, in order to provide further insights into the relationships among epigenetic features and a stressful condition in chickens (*Gallus gallus*).

**Results:**

Chickens raised under control conditions (n = 22) and chickens exposed to a social isolation protocol (n = 24) were used to identify differentially methylated regions (DMRs). A total of 60 DMRs were selected by a threshold, after bioinformatic pre-processing and analysis. The treatment was included as a binary variable (control = 0; stress = 1). Thereafter, a BN approach was applied: initially, a pre-filtering test was used for identifying pairs of features that must not be included in the process of learning the structure of the network; then, the average probability values for each arc of being part of the network were calculated; and finally, the arcs that were part of the consensus network were selected. The structure of the BN consisted of 47 out of 61 features (60 DMRs and the stressful condition), displaying 43 functional relationships. The stress condition was connected to two DMRs, one of them playing a role in tight and adhesive intracellular junctions in organs such as ovary, intestine, and brain.

**Conclusions:**

We clearly explain our steps in making each analysis choice, from discrete BN models to final generation of a consensus network from multiple model averaging searches. The epigenetic BN unravelled functional relationships among the DMRs, as well as epigenetic features in close association with the stressful condition the chickens were exposed to. The DMRs interacting with the stress condition could be further explored in future studies as possible biomarkers of stress in poultry species.

**Supplementary Information:**

The online version contains supplementary material available at 10.1186/s12859-022-04800-0.

## Background

Understanding biological systems, from molecular and cellular interactions to ecological relationships between species and the environment, can be a very difficult, complex and challenging task [1–5]. Computational biology combines computer science techniques applied in a wide range of biological fields with the aim of discovering or unravelling hidden information in biological systems [1, 6]. In the particular field of genetics and epigenetics, improvements in technology and the development of methodological tools now enable hundreds of thousands of genetic/epigenetic markers per individual together with the identification of genetic or epigenetic features of interest [1, 6]. The relationships among these genetic or epigenetic features can be explored by building probabilistic networks. This allows the inclusion of particular conditions (e.g. sex, domesticated phenotypes or a treatment) into the model [7–9]. Bayesian networks (BNs) are a type of probabilistic network that have been applied to many biological systems such as ecology, proteomics, and genomics, in order to model the dependencies among a set of given features [3, 4, 8, 10–12].

BNs are graphical models that represent joint probability distributions of a given set of variables [13]. They are directed acyclic graphs (DAGs), consisting of a set of nodes, which represent the variables, and a set of arcs or edges, representing the relationships among nodes [7, 13, 14]. BNs are based on probability theory; therefore, considering a given set of variables and a DAG, the following formula can be used to describe the network [12]:

$$\Pr \left( {X_{1} ,X_{2} , \ldots ,X_{n} } \right) = \mathop \prod \limits_{i = 1}^{p} {\text{Pr}}(X_{i} |Pa_{i} )$$

where each $${X}_{i}$$ represents one of the variables, and $${Pa}_{i}$$ is the parents of $${X}_{i}$$ (nodes with outgoing arcs to the variable $${X}_{i}$$) [7, 13, 14]. The probability of a certain variable $${X}_{i}$$ is dependent on the values of its parents ($$P{a}_{i})$$ [7, 13, 14]. Focusing on a particular variable, parents are defined as those nodes whose arcs are incoming to the variable, children are defined as those nodes whose arcs are outgoing from the variable, and spouses are defined as those nodes that share a common child (or children) with the variable. The set of parents, children, and spouses is one of the main properties of BNs, known as the Markov Blanket. This property makes the node of interest completely independent from the rest of the variables that do not belong to the Markov Blanket [15].

The structure of BNs can be learned through application of BN algorithms to measured data: this is a form of unsupervised learning, revealing patterns in the data. This can be helpful in genetics and epigenetics to discover potential pathways and highly connected nodes as features of interest [8, 16]. Additionally, the Markov Blanket property of a particular condition, stress in this study, could be useful for identifying potential biomarkers or target genes associated with the condition [17, 18]. While there has been much use of BNs to study relationships among genetic variables, there is much variation in software applied, heuristic search choices, scoring metrics, and construction of a ‘solution’, among others [19–25], and little guidance about how to navigate this array of options given features of a particular dataset. Many analyses either say what was done without providing reasoning behind choices and/or replicate methods of previous work.

The aim of our study was two-fold: first, to apply a BN structure learning approach in order to provide further insights into the relationships between epigenetics and induced stress in a poultry animal model, the chicken (*Gallus gallus*); and second, to clearly lay out our decision-making process in order to provide a roadmap to enable others to make principled choices when undertaking BN analysis. The implementation of our approach will bring further light into the stress phenomenon in poultry science by discovering potential hallmark epigenetic features related to a stress condition together with the possibility to hypothesise and to design future studies based on the findings. Additionally, laying out the steps taken as well as the decisions made as a novel analytical pathway will allow other researchers to implement our approach in their own datasets, to unravel informative interactions and relationships between genetic and/or non-genetic variables. The focus of these aims is to create novel approaches for hypothesis building considering genomic (particularly epigenomic) data.

## Results

### Bayesian network decisions

**Fig. 1:**
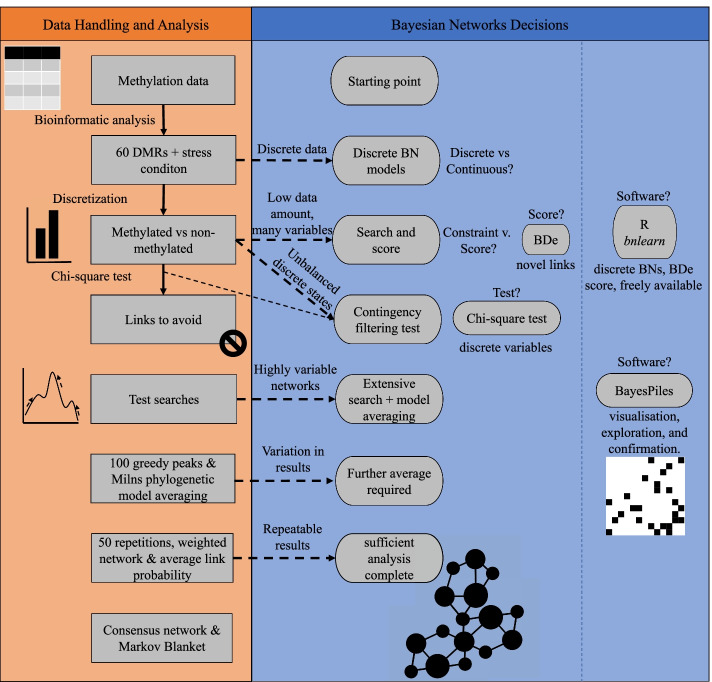
Steps taken and decisions made to build a consensus Bayesian network. The starting point was methylation data from 46 chickens under two treatment conditions (22 control, 24 stress). Bioinformatic analyses were performed as described in [54, 57]. Thereafter, a set of 60 differentially methylated regions (DMRs) were selected. The corresponding methylation values of each DMR were counts (ranging 0–39). Considering that the most frequent value was 0, binary discretization was implemented, leading us to explore discrete Bayesian network (BN) algorithms: we used the *bnlearn* package in R, exploring the search space with a score-and-search algorithm and the BDe score. Considering that the data had imbalances between binary states that could lead to the discovery of artefactual arcs, a contingency test (chi-square) was applied to all possible pairs of variables to create a list of arcs to avoid. Test searches and the software BayesPiles showed that the search space was complex and building the consensus Bayesian network required a strategic and iterative approach: the combination of a phylogenetic model averaging, plus further selection of arcs common to all searches into a consensus weighted Bayesian network

Figure 1 provides an overview of the Bayesian network decisions made alongside corresponding analysis steps.

A first major choice in BN analysis is whether to use discrete versus continuous models: this refers to the form of the analysed data, whether they are provided as continuous values or discrete states, which can be ordinal (e.g., present/absent, low/medium/high) or have no order (e.g., red/green, a sampling location). However, while discrete data in general requires use of a discrete BN model, continuous data does not: continuous measured values can be ‘discretised’ into ordinal states. When one has continuous data, a decision needs to be made. Continuous BNs make use of the numeric value of measured variables, capturing the full range of values, but are restricted to additive interactions; discrete BNs use discrete categories for variable values, potentially losing information, but allow for combinatoric interactions (e.g., requiring both parents to be present) [13]. Our data (see “Methods” Section) consisted of 60 differentially methylated regions (DMRs) identified when comparing methylomic profiles in red blood cells between two experimental conditions, controls (22) vs stress (24) in 46 male White Leghorn chickens (*Gallus gallus*): these represent the features in our dataset. The experimental condition was a discrete variable. These experimentally identified DMRs allowed us to localize genomic regions from which reads were extracted per individual analysed. For the purpose of the analyses performed here, these regions were also named as DMRs. These DMRs were integer values representing the number of sequenced reads for each individual, which represents the methylation level of that specific region per individual; however, the value of 0 (no methylation) was by far the most common, therefore, meaningful discretisation into no-methylation and methylation was a sensible choice. This discrete data combined with the ability of discrete BNs to represent combinatoric interactions, which may be expected in genetic systems [26], led us to choose discrete BN models.

There are two major branches of BN discovery: constraint-based and search-based. Constraint-based methods use conditional independence tests to eliminate network structures that are inconsistent with discovered conditional independence relationship, returning a network solution which fits these constraints. Search-based algorithms perform a heuristic search through network structures, selecting structures with high scores under a specific scoring metric. Constraint-based methods can be sensitive to node order, returning different structures with different ordering of the variables of the used data file [27] and are often considered less accurate than score-based methods [28] (although this has been brought into question in recent years [29]). Search-based algorithms can produce variable answers due to randomness within a heuristic search, but this is not dependent on node order in the data file. Thus, the same dataset can be queried multiple times for capturing a range of solutions. Search-based methods provide a score representing the probability of a returned solution: this can be used in a principled way to combine multiple different answers, weighting networks by their probability [3]. This feature is particularly useful in a situation like ours, with low data amount and high number of variables, where combining results from multiple searches can provide greater confidence in an answer. Additionally, score-based algorithms are more commonly used and there is a larger variety tools available [30]. Because of their wider use, tool availability, and relatively easy option to develop further methodology combining multiple network solutions, we chose to apply search-based algorithms for our roadmap.

Given discrete BNs, there are a number of scoring metrics to choose among. We wished to maximise our ability to find novel connections, thus we selected the Bayesian Dirichlet equivalent (BDe) score [13], which has been shown to be less conservative than others (Bayesian Information Criterion [BIC] and Mutual Information [MI] [31]). Additionally, while our 46 datapoints are on the low side for recovering Bayesian networks [31], this data amount is sufficient to recover up to three parents per node using the BDe score [32], which generates a reasonably complex network. It has been shown for the BDe score that lower data amounts result in recovery of fewer arcs, but does not result in erroneous arcs [31]. Thus, we can remain confident in those arcs we do recover.

Another choice to make is what software to use to perform the Bayesian network analysis, with options ranging from coding it oneself [19, 20] to a variety of free and proprietary platforms [21–25]. This choice can be somewhat arbitrary, as the underlying theory remains the same, but will be constrained by one’s analysis choices, in our case discrete networks using a BDe score. We elected to use the R package bnlearn [33], as free, open-source software which had our desired functionality.

**Fig. 2:**
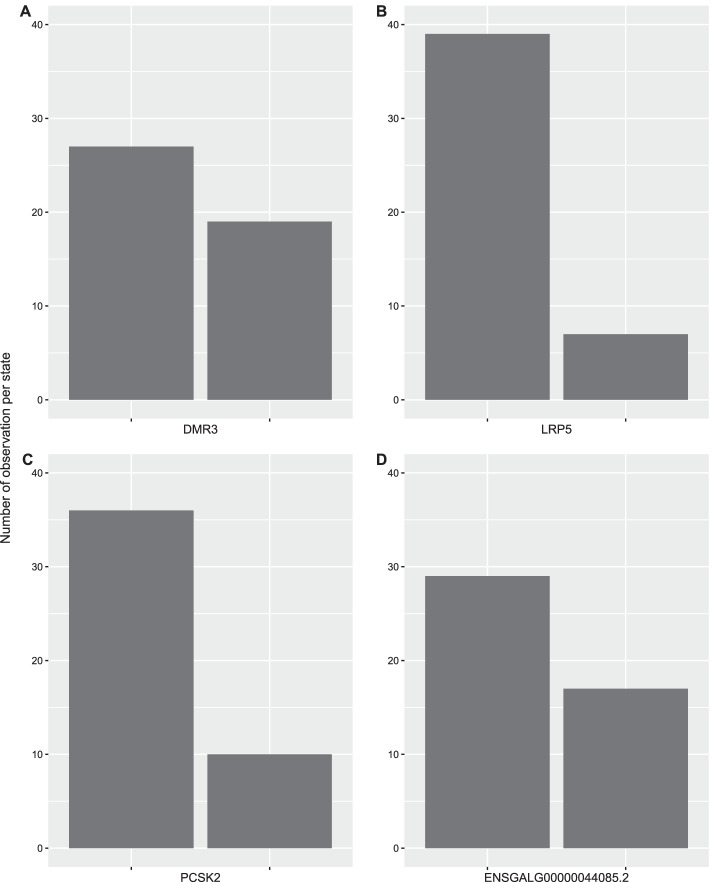
Distribution of four of the differentially methylated regions (DMRs) once a binary discretization method was applied. The state 0 represents values with absence of methylation, the state 1 represents values with presence of methylation. These four DMRs (**A**–**D**) are representative of imbalances between the two states, as zero was the most popular state among different DMRs

Finally, choices regarding the search process must be made. In order to make informed decisions, iterative exploration of the data and initial search results is required. First, we examined our discrete data, and found that there was an imbalance in discrete states for many of the DMRs (more no-methylation, Fig. 2). Because such imbalanced states can create artefactual connections by overrepresented states appearing to be good predictors of each other, regardless of the presence of the rarer states [3], we applied the method of contingency test filtering from Milns et al. [3]: we applied pair-wise chi-square tests, identifying those pairs of variables with chi-square p-values equal to or greater than 0.25 as showing no potential dependence. These were provided to the BN as a list of arcs that must not be considered in the process of building the network [3]. In total, contingency test filtering identified a total of 960 arcs (of the 3,660 possible arcs) to avoid.

**Fig. 3:**
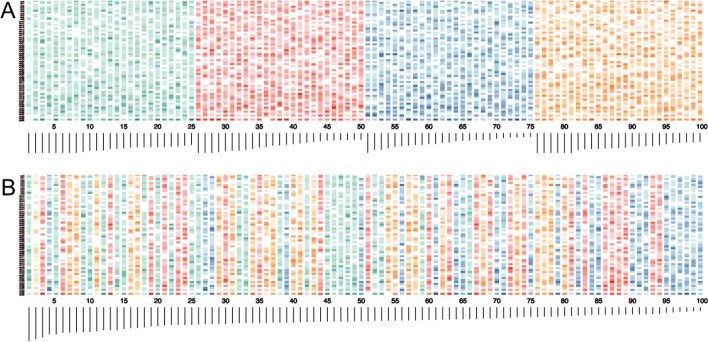
BayesPiles investigation of search space. Top networks found from four separate collections of searches, representing peaks of many different hills in the search space. BayesPiles visualises a summary of network structure as a shaded stack representing out-degree of each node (darker = higher) above a bar representing network score (longer = higher), with networks along the x-axis and nodes along the y-axis. **A** shows the highest 25 networks for four collections of searches (different colours), with highest network to the left. The strong variation in network structure (different patterns in the shaded bars) indicates that these networks are tops of different peaks in the search space, not the final climb of a single hill. **B** shows the final 25 networks from all four searches combined, sorted by their score. The mixing of colours throughout shows the high variation in search peaks: each collection of searches explored different areas of the search space, finding different high-scoring structures

We preformed initial heuristic searches using the bnlearn R package, finding a large variety in network structure, suggesting that extensive search and model averaging would be the best approach. We confirmed this with analysis via BayesPiles [34], which showed highly variable top networks across different searches (Fig. 3). Networks similar in score varied strongly in structure. This variation indicates that the top networks found are in different areas of the search space, and not simply fine variations of one general area. Thus, we elected to apply the modelling averaging approach from Milns et al. [3], which has been shown to produce similar sets of highly probability arcs from different collections of top networks [3]: we performed 100 greedy hill climbs (see “Methods” Section) from 100 random starting networks, and applied the Milns model averaging approach to identify highly probable arcs [3]. As there was still some variation even in these highly probable arcs, we repeated this process 50 times, selected those arcs common to all searches, and took the average probability of the common highly probable arcs across all repetitions, to produce a final consensus network. Repetition of this analysis showed repeatable results, identifying the same top relationships between DMRs and the same Markov Blanket of the stress condition, thus we determined this was sufficient exploration of the search space.

### Discovered Bayesian network structure

**Fig. 4:**
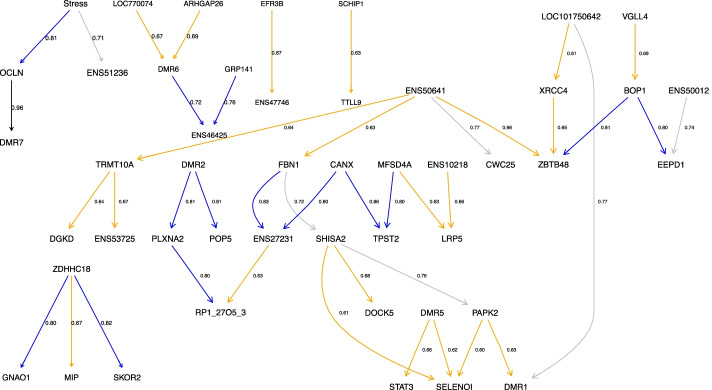
Consensus network of DMRs. Networks were built with common arcs to 50 searches, each one of these searches consisted of a starting point of 100 random graphs. Features representing the differentially methylated regions (named by related gene or region, see “Methods” Section) and the stress conditions are nodes; lines between nodes represent the identified relationships. Arc labels represent the average probability of belonging to the consensus network, the higher the values, the higher the probability. Different colours represent different ranges of probabilities: black: 0.90–1.00, blue: 0.80–0.89; grey: 0.70–0.79; orange: 0.60–0.69

A total of 43 arcs were common to all 50 searches. These arcs and their average probability values of being part of the top 100 networks are shown in Additional file 1, and the consensus network built with these arcs is shown in Fig. 4. The consensus network included 47 out of the 61 features (60 DMRs plus experimental condition). Among these arcs, relationships between DMRs OCLN—DMR7 (distal intergenic region, see “Methods” Section), CANX—TPST2, and FBN1—ENS27231 (unannotated region, see “Methods” Section) had the highest values of probabilities of being part of the consensus network (0.96, 0.86 and 0.83, respectively) (Table 1).

**Table 1:** Features of interest discovered via Bayesian network

Symbol	Gene	Identified by	Gene information
OCLN	Occludin	Markov Blanket	Intracellular tight junctions and adhesion. Limiting (allowing/preventing) the exchange of substances and/or cells
CANX	Calnexin	Highest probability values	Role when unfolded/misfolded proteins exceed the capacity of chaperones. Functionality associated with the resistance of the blood brain barrier
FBN1	Fibrillin-1	Highest probability values	Component of microfibrils which make up extracellular matrix protecting cells, including nerve cells
TPST 1 / TPST 2	Tyrosyl protein sulfotransferases	Highest probability values	Both proteins are in charge of the correct functioning of PSLG-1 (P-selectin), which promotes adhesive interactions with other selectins that might lead to inflammatory diseases
ENS51236	None	Markov Blanket	Function yet unknown
ENS27231	None	Highest probability values	Function yet unknown
DMR7	None	Highest probability values	Function yet unknown

The DMR name (see “Methods” Section) as shown in the network in Fig. 4 is provided in the ‘Symbol’ column; the gene name (if existing) in ‘Gene’, and the remaining two columns represent how we identified this feature of interest from the network structure and information about gene function, respectively

The application of the Markov Blanket property in order to get the set of parents, children, and spouses in close relationship with the treatment identified only two DMRs, OCLN and ENS51236 (unannotated region, see “Methods” Section; Table 1). The arc between stress and OCLN had the highest average probability value (0.81).

## Discussion

Behind biological systems lies a series of complex and intricate relationships among features [2, 3]. The application of BNs can be a useful approach to discover, identify and unravel hidden patterns within the data, and gain insights into a biological area of knowledge [10, 16]. However, there is little practical guidance for how to make choices among the array of possibilities within a BN analysis. Here, we have undertaken a practical application of BNs to a particular question in poultry epigenetics, while clearly stating our analysis choices. We explained our reasoning behind using a discrete, rather than a continuous, BN due to the distribution of our data, how we chose the BDe score, and the software applied. We explained our analysis of our dataset's discrete states and the choice to use chi-square contingency-test filtering to avoid artefacts from imbalanced discrete states. We showed our exploration of the search space structure for our question, including using the specialised software BayesPiles [3], which revealed the space to be highly varied and thus to require complex model averaging techniques. We applied techniques developed in a similarly varied search space [3], and added further refinements of combining multiple searches. We hope that our clarity surrounding our choices will provide a roadmap for others beginning a BN analysis.

The Markov Blanket of the stress condition together with those DMRs showing the highest probability values of being part of the consensus network appear related to the functional structure of the brain and a possible link with the immune system. Starting with OCLN, Occludin, this gene showed the highest probability value and it belonged to the Markov Blanket of the stress condition. Occludin is a gene whose major functionality is associated with intracellular tight junctions and adhesion, defining a selective barrier and limiting the exchange of substances and/or cells in different tissues such as the chicken ovary, the chicken intestinal mucosa, or the human brain [35–39]. In the chicken ovarian follicles, Occludin plays a role in allowing or preventing the exchange of yolk material, especially during the first stages of the formation of the follicles, considering that the expression values were increased [35, 39]. In case of the brain, Occludin and other genes are involved in the permeability of the blood brain barrier, as its integrity is crucial for the correct functioning of the central nervous system [37, 38]. In patients suffering from a fatal heat stroke, increased expression values of OCLN were found, and authors suggested that it could be aimed at restoring junctional complexes and the barrier function as a compensatory mechanism [38]. Considering that the stress response is initially triggered in the central nervous system, it is possible that OCLN is playing a key role protecting the integrity of the blood brain barrier to prevent any nervous disfunction, that would be crucial when dealing with the influence of a stressor.

The arc between CANX and TPST 2 was among the arcs with the highest probability values. The biological functionality of CANX, Calnexin, can be divided into two major categories as it is linked to the immune system as well as to the blood brain barrier [40–43]. Chickens inoculated with *Salmonella* Enteritidis as an immune challenge increased the abundance of Calnexin in heterophils (a subpopulation of leukocytes) [40]. Together with other proteins, Calnexin belongs to the endoplasmic reticulum proteins and their functionality comes into play when the unfolded or misfolded proteins exceed the capacity of chaperones or when the luminal conditions are not optimal for the correct processing of new proteins [42]. Regarding Calnexin functionality in the brain, Jung et al. [43] found that this gene plays a major role in multiple sclerosis and its equivalent in mice, as the loss of CANX increased the resistance of the blood brain barrier, avoiding the infiltration of cells belonging to the immune system and the induction of inflammation markers [43]. The other DMR interacting with CANX was TPST 2, a tyrosyl protein sulfotransferase that, and together with TPST 1, are in charge of the correct functioning of P-selectin glycoprotein ligand-1 (PSGL-1) by transferring tyrosine residues [41, 44, 45]. PSGL-1 is expressed on leukocytes and promotes binding and adhesive interaction with other selectins that may lead to inflammatory disorders as a consequence of a potential pathological recruitment of leukocytes [46].

Among the Markov Blanket as well as the arcs with the highest weight values there were 3 DMRs whose function and/or annotation is still yet unknown (DMR7, ENS51236, and ENS27231). Our finding highlights two different advantages of implementing BNs: on the one hand, studies focusing only on bioinformatic analysis would generally ignore these DMRs or genes, because the functionality of them will not be found in sources such as KEGG pathways or GO terms. On the other hand, the power of BN algorithm discovered novel markers that might be worth exploring, for example how ENS27231 might interact with FBN1 and relationship of the extracellular matrix with stress response. Learning the structure of a BN with a set of highly significant genetic features can be the starting point of future research. Instead of focusing on the bigger picture that bioinformatic studies provide, analysis of only a reduced number of features would be more accurate to gain a further insight into the stress phenomenon.

The stressful condition, in this particular study, was directly connected to only one epigenetic feature, OCLN, while the Markov Blanket consisted of two epigenetic features. It is then plausible to ask whether these two DMRs can be explored as biomarkers of stress in chickens. Even though our approach was mostly exploratory using a relatively small number of observations, this did not prevent implementation of BN algorithms; however, these small number of observations might have had an impact on the search space, requiring all the steps taken throughout this study. Additionally, the BDe score, used in our approach as the score to find the networks that best fitted the data, has previously been shown to have a better performance compared to other scores, such as the BIC score, when dealing with small number of observations. In this scenario, the BDe score is considered to be less conservative, being able to identify arcs between discrete variables, while the BIC score could not recover any of the arcs [24]. Considering our finding as the starting point, future studies can be designed with the aim of evaluating the expression and/or methylation patterns of only these two genetic features under two experimental conditions, non-stress and stress. Thereafter, knowledge can be transferred into other fields such as animal welfare and poultry production. For example, one of the main principles of animal welfare is the absence of distress in association with a comfortable environment [18, 47, 48]. Stress can be highlighted as one of the major problems faced by the poultry industry nowadays, and the knowledge discovered by BNs can be further used to develop breeding protocols and genetic lines [49, 50]. Even though in this particular study the condition was stress, it is important to mention that the condition could be of any other nature, such as gender, male vs female; phenotypes, ancestral vs domesticated chickens; or even different stages in life, juvenile vs adult [51–53]. In this context, the approach implemented in this study can be applied in genetics and epigenetics as a first approximation to gain basic knowledge in regard to a particular condition, with potential implications in applied science.

## Methods

### Dataset

The data was accessed and downloaded from the European Nucleotide Archive (ENA, www.ebi.ac.uk), under the accession number PRJEB34868 [54]. The dataset consisted of 46 male White Leghorn chickens (*Gallus gallus*). The experiment involved 0–26 days aged chickens, 22 raised under control conditions, while the other 24 were exposed to a social isolation protocol. This isolation protocol was applied from the day 4 of age until the day 26 of age (period of 21 continuous days), as described by Pértille et al. [54]. Briefly, birds under the stressful condition were daily exposed to social isolation for one hour during the first week, two hours during the second week, and three hours during the third and final week. During the exposure to the isolation stress, birds were individually placed in a box with vocal but no visual or physical contact with other birds. Thus, during the stress treatment, birds were exposed to a combination of stressors: social isolation and deprivation of food and water [54, 55]. The control animals were not exposed to the social isolation protocol, but they were raised under the same environmental conditions as the stressed birds. The identification of differentially methylated regions (DMRs) between these experimental groups included a series of steps such as blood collection at day 26 of age (2 h after the last day of isolation was ended) in order to extract the DNA from red blood cells, the preparation of the libraries using the GBS-MeDIP method [56] to sequence the DNA fragments and finally the bioinformatic pre-processing and analysis to identify the DMRs [54, 57]. The DMRs identified in this study were selected by first defining ‘Regions of Interest’ (ROI) showing differences in sequencing coverage between the treatment and control groups. This was done with MACS2, which is a recommended tool to identify sample-wise ‘peak specific’ methylated regions of variable sizes in experiments using paired controls to determine enrichment against background [58–60]. Then, we applied the weighted trimmed mean of M-values (TMM) method within edgeR on these ROI obtained with MACS2. TMM is used to calculate scale factors between libraries. One of the standard outputs of this edgeR test is a p-value (edgeR.p.value). Based on this, 60 DMRs were selected with *p* ≤ 0.005. DMRs were annotated and divided into 4 different categories based on the features of the genome in the region: promoter, distal intergenic, intron, or exon. DMRs categorised as promoters, introns and exons were annotated with the corresponding gene name. Promoters, introns, and exons without a proper gene name were assigned their corresponding ENSEMBL gene name using the first three letters and the numbers after the zeros (e.g. ENS50641 represents ENSGALG00000050641.1). DMRs annotated as DMR1 up to DRM7 correspond to distal intergenic regions without a proper gene name. A list of the 60 DMRs used and their annotations is provided in Additional file 2. 

### Data discretization and contingency test

The DMR dataset (46 samples and 60 variables) consisted of individual counts obtained within the experimentally obtained DMRs described above, corresponding to the number of segments aligned to a particular DNA region, values ranging from 0 to 39. With the data already pre-processed, our initial step to build the consensus BN was to further discretize this count data with the aim of filtering noise as well as increasing the statistical power [31]. The most statistical power is provided by all discrete states having roughly equivalent numbers of data points [3, 13]; here, zero counts was the most abundant observation, and thus the closest to this ideal was a binary dataset with two categories: zero and one. All original values equal to zero were assigned a new value of zero (no methylation), while the rest of the values were assigned a new value of one (methylation). In addition to the DMRs, the stressful condition was included in the dataset as a binary variable, considering the control condition as 0 and the stress condition as 1 (22 individuals = 0, 24 individuals = 1). The DMRs plus the stressful condition are our features which are included as nodes in the network. An overabundance of the discrete state of zero remained. An imbalance of discrete states can lead to potential artefacts where high-frequency states of different variables overwhelm the BDe scoring matrix and appear to predict each other, irrespective the distribution of lower-frequency states [3]. In order to combat this artefact, contingency tests can be applied to filter out any pairs of variables showing no evidence of contingency with each other (e.g., an arc between them would be more likely to be an artefact) [3]. Thus, we applied pair-wise contingency-test filtering as in Milns et al. [3]: a chi-square contingency test was applied to all pairs of variables, using a p-value of 0.25 as the cut-off point where we considered there to be no evidence of contingency. Thus all pairs of variables with a chi-square p-values 0.25 or above were filtered out as showing no possible dependence between them [3]. These were included in the Bayesian networks analysis as a list of arcs to be blocked, representing prior information that these arcs should be excluded from the network [12, 61].

### Bayesian network analysis

The R package “*bnlearn”* [33] was used to learn the structure of the network. Initial tests were done by starting groups of 100 searches from random graphs generated by the *random.graph* function, using *tabu* search function, with the BDe score and the list of arcs to be blocked included [12, 61]. Summary networks of arcs found across these groups of searches were analysed for arc correspondence and showed high variability. Variability in search results was confirmed using BayesPiles [34], which requires use of the Banjo software [62]: equivalent settings using the BDe score and a greedy (closest available to tabu) search were set in Banjo, and the list of arcs to be blocked included. Four sets of searches including multiple starts from random networks were visualised (Fig. 3), revealing again high variability.

Thus, we decided to use a method previously applied in an ecological system with a similarly high variability in search results [3]. This method collects top networks from multiple searches (100 searches both in [3] and here), then applies a phylogenetic model averaging approach considering the score of the network to develop probabilities of arcs being in a high-scoring network. These probabilities are clustered into higher and lower probability clusters, and are provided uncertainty values for cluster membership. Those arcs in the higher probability cluster (with a probability and uncertainty cut-off) are presented as the final network. To perform this analysis, we started 100 searches from random graphs generated by the *random.graph* function, using *tabu* search function, with the BDe score and the list of blocked arcs included, as above, identifying 100 top networks. This search process took approximately 4 min on a Mac laptop running OS 12.1. The arcs present in the 100 top networks, along with the network scores, were input into the function *relationshipProb* developed by Milns and collaborators [3], which provides an average probability for each arc. These probabilities were then input into their *makeclustersIDhigh* function, which estimates the probability of each arc being part of one out of two categories: low probability or high probability. Each arc was assigned to either a low probability or high probability category in addition to a value corresponding to the uncertainty associated with the classification process [3]. The arcs considered as highly probable functional relationships were selected with probability values greater than or equal to 0.5 and an uncertainty value equal or lower than 0.01. This model averaging and identification of highly probable relationships took approximately 15 s on the same machine. Additional files 3 and 4 provide the R code and the data, respectively, for these BN analyses.

This process still resulted in more variation than desired, thus in order to build a consensus network, the arcs common to 50 repetitions of the above process (starting point of each search, 100 random graphs, then application of the Milns et al. [3] method to identify highly probable functional relationships) were combined. For each arc common to the 50 repetitions, an average value of the probabilities was calculated and used for building a weighted network. The Markov Blanket of the treatment was identified by applying the *mb* function within the *“bnlearn”* package.

## Supplementary Information

**Additional file 1.** Arcs and their corresponding probabilities of being part of a high scoring network.List of arcs identified between differentially methylated regions and with the stress condition, with their corresponding probabilities of being part of a high scoring network. The first column (“arc”) is an arbiratry numbering for the arc;the second column (“from”) represents the parent node for each arc (arcs from); the third column (“to”) represents the child node for each arc (arcs to); the third column (“Average.Probablity”) represents the average probability value for each arc of being part of a high scoring network.

**Additional file 2.** Differentially methylated regions and their annotations. List of differentially methylated regions (DMR) with their corresponding genetic annotation terms. The first column (“SYMBOL”) represents the abbreviated gene name of the methylated region; those which say “annotated” plus a number means that the symbol for that particular DMR was not available; the second column (“Gene ID”) represents the ENSEMBL gene ID; the third column (“Description”) represents the description of the DMR (NA for those not available); the fourth column (“Chromosome””) represents the chromosome in which the DMR is located; the fifth column (“Location”) represent the location in the chromosome of each particular DMR (chromosome number repeated); the seventh column.

**Additional file 3.** R code for data discretisation, Bayesian network generation, and model averaging.R code is provided, that operates with Additional file 4 (the DMR raw data), to discretise into presence/absence discrete states, run 100 Bayesian network greedy searches for input into the model averaging, and perform the model averaging to generate a file containing the highly probable arcs. Comments direct the reader to how to use this code on their own data set.

**Additional file 4.** Differential methylated regions plus treatment raw data.The raw methylation values for each DMR (number of sequenced reads) plus treatment representing stress condition (0 = control, 1 = stress) are provided in tab-delimited format. The header row contains DMR name or ‘Treatment’; rows are individuals.

## Data Availability

The datasets analysed during the current study are available in the European Nucleotide Archive (ENA) repository, under the accession number PRJEB34868: http://www.ebi.ac.uk/ena/data/view/PRJEB34868.

## Abbreviations

BDe: Bayesian Dirichlet equivalent
BIC: Bayesian information criterion
BN: Bayesian network
CALX: Calnexin
DAG: Directed acyclic graph
DMR: Differentially methylated region
ENA: European nucleotide archive
MI: Mutual information
OCLN: Occludin
TMM: Trimmed mean of M-values
TPST: Tyrosyl protein sulfotransferase
ROI: Regions of interest
